# Exploring *Chlamydia trachomatis* screening in Shenzhen: a cost-effective sample pooling strategy for pre-marital and pre-pregnancy populations

**DOI:** 10.3389/fpubh.2025.1697573

**Published:** 2025-12-05

**Authors:** Li Zhang, Yannan Xu, Hailing Ye, Yu Xie, Lishan Tian, Qiuhong Wu, Yi Ding, Tao Zhang, Lifeng Tong, Xiangsheng Chen, Jun Yuan

**Affiliations:** 1 Shenzhen Nanshan Center for Chronic Disease Control, Shenzhen, Guangdong, China; 2 School of Public Health, Shantou University, Shantou, Guangdong, China; 3 Department of Epidemiology and Health Statistics, Xiangya School of Public Health, Central South University, Changsha, Hunan, China; 4 National Center for STD Control, Chinese Academy of Medical Sciences and Peking Union Medical College Institute of Dermatology, Nanjing, Jiangsu, China

**Keywords:** *Chlamydia trachomatis*, pooling test, cost, polymerase chain reaction, pre-marital and pre-pregnancy populations

## Abstract

**Objectives:**

This study evaluates the feasibility of a sample pooling strategy for *Chlamydia trachomatis* detection in pre-marital and pre-pregnancy screening in China, where pooled testing for CT remains limited despite its use in COVID-19 and HIV screening.

**Methods:**

From January to May 2024, 8,142 urine samples were collected from participants in pre-marital and pre-pregnancy health checks in Nanshan District. Positive urine samples with different Ct value ranges were selected, and negative urine samples were used as diluents to simulate mixed samples. We compared the sensitivity, specificity, and other metrics between the pooling tests and individual tests. A paired t-test was used to assess the statistical significance of the mean ΔCt values.

**Results:**

The 6-sample pool (1 positive:5 negative) demonstrated high sensitivity (96.7%; 95% CI: 94.1–99.3%) with a mean Ct value of 35.6 (range: 33.6–37.4). The specificity was 100% (3/3), PPV was 100% (175/175), and NPV was 33.3% (3/9). The total agreement with single testing was 96.7% (178/184; 95% CI: 94.2–99.3%). The mean ΔCt value between single and pooled tests was 2.8 (range: 2–3.2), with a statistically significant difference (*p* < 0.05).

**Conclusion:**

The 6-sample pooling strategy demonstrated acceptable sensitivity (96.7%) and significantly improved screening efficiency, suggesting its potential utility for large-scale pre-marital and pre-pregnancy screening programs in appropriate settings.

## 1 Introduction

*Chlamydia trachomatis* (*C. trachomatis*) can cause sexually transmitted diseases (STDs), primarily characterized by genital inflammation. It is one of the five key STDs in China (1). In 2020, there was an estimated global prevalence rate of 4.0% among women and 2.5% among men (2). From 2015 to 2019, the reported incidence rate in China increased at an average annual growth rate of 10.44%. Guangdong province has been identified as a region with a high incidence of *C. trachomatis* infection (3). *C. trachomatis* infections can result in pelvic inflammatory disease (PID) without therapy, an increased risk of infertility and ectopic pregnancy in women, as well as painful testicular infections in men (2, 4). To effectively control *C. trachomatis* infections, some developed countries now recommend annual screening for *C. trachomatis* infections in women of childbearing age (5). In addition, a cost-effectiveness analysis of *C. trachomatis* screening programs revealed that screening for chlamydia infection in women of childbearing age (15–30 years) is economically viable and offers considerable health economic advantages (6).

To significantly enhance screening efficiency, pooling test is often adopted as a more effective approach for urgent and large-scale infection screening programs. The pooling test strategy involves combining samples from multiple individuals and analyzing them collectively as a single group (7), aiming to minimize the number of tests performed on a set of samples by using the ability to merge subsets of the test samples. The basic principle is to mix n samples (e.g., urine, throat swabs) into a single sampling tube for a single uniform nucleic acid test. If the result of that tube is negative, it means that all the sampled persons are negative; if it is positive, all the sampled persons in that tube are immediately retested in a single person and a single tube in order to find out which of them are positive (8). In 1943, economist Robert Dorfman pioneered pooled testing theory to detect syphilis in WWII US soldiers (9). The pooling strategy was then gradually and widely used to detect pathogens such as coronavirus disease (COVID-19) (10), HIV and hepatitis C viruses (11, 12). Meanwhile, sample pooling test for *C. trachomatis* have been conducted in various countries. For instance, India, Australia, and Canada have employed screening methods utilizing 5-mix, 5-mix, and 4-mix, respectively (13–15). The utilization of the pooling strategy has been demonstrated to markedly enhance the efficacy of screening procedures whilst concurrently reducing the financial burden associated with such tests.

Currently, the use of pooling tests for the detection of *C. trachomatis* has not been extensively explored in studies conducted in China. The efficacy, sensitivity, and specificity of the pooling strategy in different subjects remains to be validated. Therefore, this study will deeply explore the feasibility of mixed nucleic acid polymerase chain reaction (PCR) in pre-marital and pre-pregnancy (PMPP) population to provide a rapid and cost-effective pooling test strategy for large-scale screening of *C. trachomatis*.

## 2 Methods

### 2.1 Sample sources

The source of the urine sample was the population who participated in the PM/PP health check in Nanshan District, Shenzhen, between January 2024 and May 2024. All methods were performed in accordance with the “Technical Guidelines for the Registration of Nucleic Acid Detection of *C. trachomatis* and/or *Neisseria gonorrhoeae*” and relevant national/international regulations. Written informed consent was obtained from all participants prior to sample collection, and confidentiality of personal data was strictly maintained throughout the study. Inclusion criteria:(1) males ≥ 22 years of age and females ≥ 20 years of age who had had sexual intercourse; (2) complete a structured questionnaire; (3) signing of an informed consent form; (4) not used antibiotics in 2 weeks and willingness to provide a urine sample. All of the above criteria are satisfied prior to enrolment.

#### 2.1.1 Ethical approval

The Ethical Committee of Shenzhen Nanshan Center for Chronic Disease Control (Approved No. LL20220013) approved the study. All participants signed the informed consent.

#### 2.1.2 Sample size

In this study, the sample size was calculated based on the expected sensitivity and specificity of the diagnostic method. For sensitivity, the formula used was:


$$N_{\mathrm{sen}}=\frac{Z_{1-\alpha /2}^{2}P_{\mathrm{sen}}(1-P_{\mathrm{sen}})}{d^{2}}$$

Where *Z* is the Z-score corresponding to the desired confidence level (1.96 for a 95% confidence level), *P_sen_* is the expected sensitivity (set at 95% based on preliminary data), and *d* is the margin of error (set at 0.05 for a 5% precision). Similarly, the sample size for specificity was calculated using:


$$N_{\mathrm{spe}}=\frac{Z_{1-\alpha /2}^{2}P_{\mathrm{spe}}(1-P_{\mathrm{spe}})}{d^{2}}$$

Where *P_spe_* is the expected specificity (Set at 99.5% based on preliminary data and literature review). Eventually, a total of 8,142 urine samples were collected for analysis.

### 2.2 Pre-implementation study

In anticipation of the increasing demand for testing, a pre-implementation study for sample pooling was performed with defined samples to determine the suitable pool ratio for expanded sample detection.

#### 2.2.1 Sample selection

Using the single test as standard, 11 positive samples were randomly selected, with Ct values ranging from 27.9 to 40.2. The selection process ensured the inclusion of samples with varying degrees of positivity, encompassing strongly positive (Ct ≤ 35), moderately positive (Ct ≤ 37), and weakly positive (Ct ≤ 42) samples. This approach was employed to simulate the presence of different pathogen loads in actual test samples.

#### 2.2.2 Sample pooling ratio setting

In this study, negative urine samples were used as diluents to simulate mixed samples and set up parallel tests. Equal volumes of *C. trachomatis* positive and negative samples were removed from each sample tube and placed into a new tube with a final volume of 2400ul. The sample pooling ratio is defined as the ratio of the volume of negative samples to the volume of positive samples, and the sample pool size is defined as the total number of individual samples contained in the pool. For example, for a four-mix samples pool with a pool ratio of 3, 600 uL is allocated to the positive sample and the remaining three negative samples are equally allocated 600 uL (1800 uL total). The resulting mixed urine samples of each proportion are vortexed for 60 s and then simultaneously tested on the machine with the only single positive sample of each group, with a final setting of 2–10 sample pool sizes for mixing.

#### 2.2.3 Optimal pool ratio formula (16)

*ps* = 1.24 × *P* ^-0.466^ (*ps* is the sample pool size, and *P* is the actual positivity rate).

### 2.3 Disposal of samples with discordant results

**Figure 1:**
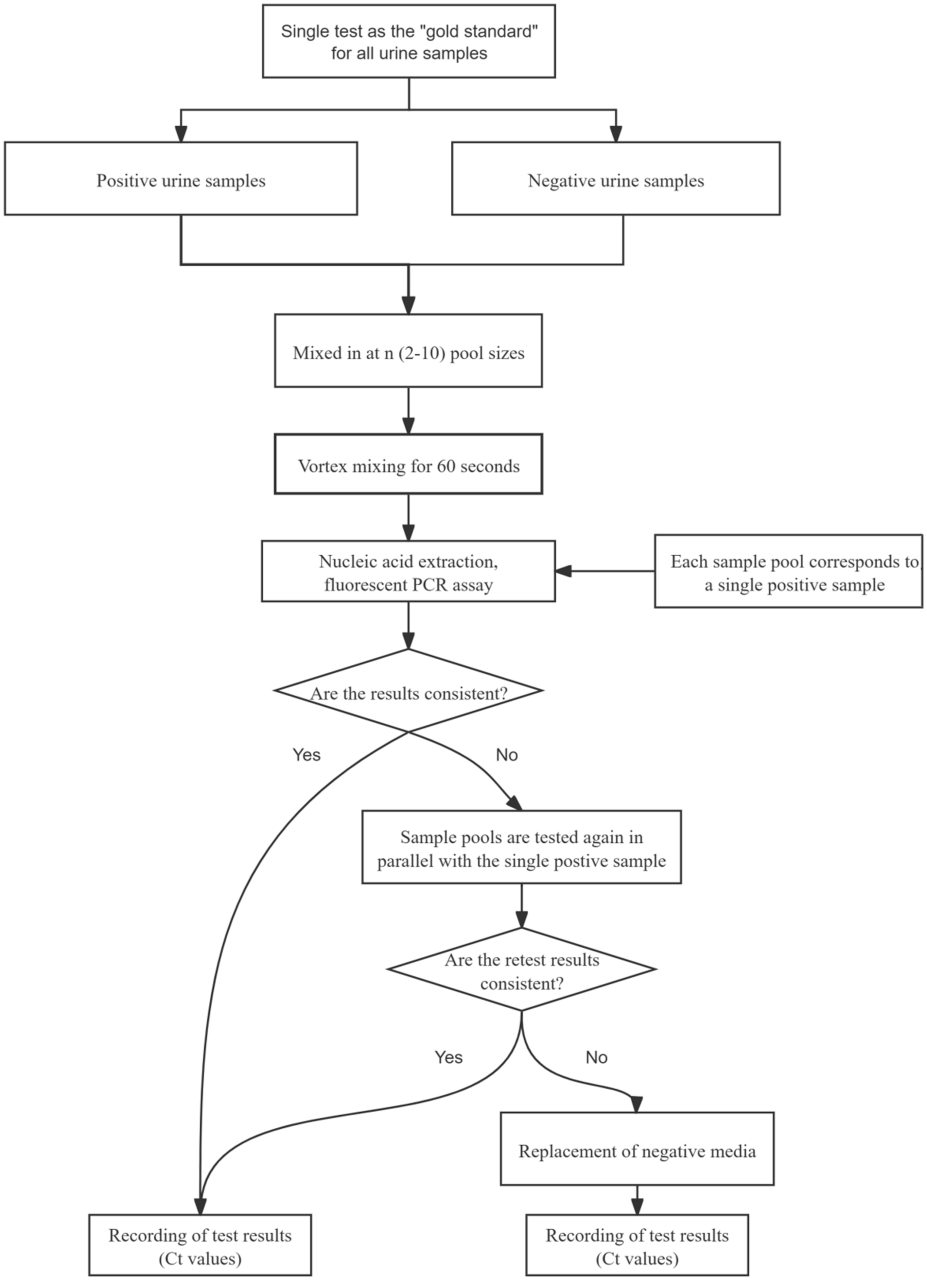
The flow of sample pool testing in this study.

In the event of inconsistent results between sample pooling and single-tube testing, both samples were retested simultaneously. If the discrepancy persisted, the samples were retested using a fresh negative medium as a replacement control, and the final Ct values were recorded to ensure result accuracy. The flow of sample pool testing in this study is shown in Figure 1.

### 2.4 Expanded sample validation

After the appropriate sample pool ratio was determined by the pre-experiment, the validation experiment was completed according to this ratio in more positive samples again following the process in Figure 1.

### 2.5 Detection methods

The Cobas 4,800 fully automated nucleic acid purification and fluorescence PCR analysis system was used in this study. The samples from the pre-implementation research and the expanded sample test were placed in the machine at the same time as the individual test samples for DNA extraction and PCR analysis, recording the Ct value.

### 2.6 Data analysis

#### 2.6.1 Statistical analysis

The mixing of one positive sample with negative ones will dilute the pathogen concentration and raise the Ct value detected by PCR. The delta Ct value (ΔCt) is defined as the absolute increase in Ct value when testing pooled samples compared to individual positive samples. Therefore, a positive Ct value represents the loss of PCR sensitivity attributable to sample pooling. Statistical significance of the mean ΔCt values was assessed using a paired t-test, *α* = 0.05.

#### 2.6.2 Evaluation indicators

The results of single and pooling test should be evaluated using appropriate indicators, such as sensitivity, specificity, and compliance rate. Assuming the purchase of 10,000 test reagents, the study used a *C. trachomatis* positivity rate of 2.3% to evaluate the economic benefits of sample pooling testing. As prevalence rises, the probability of a positive pool requiring deconstruction and further individual testing increases (17). The maximum number of assays (without QC) needed for the pooling test of 1,000 individuals was calculated using R version 4.3.3.

## 3 Results

### 3.1 Pre-experimental results

**Figure 2:**
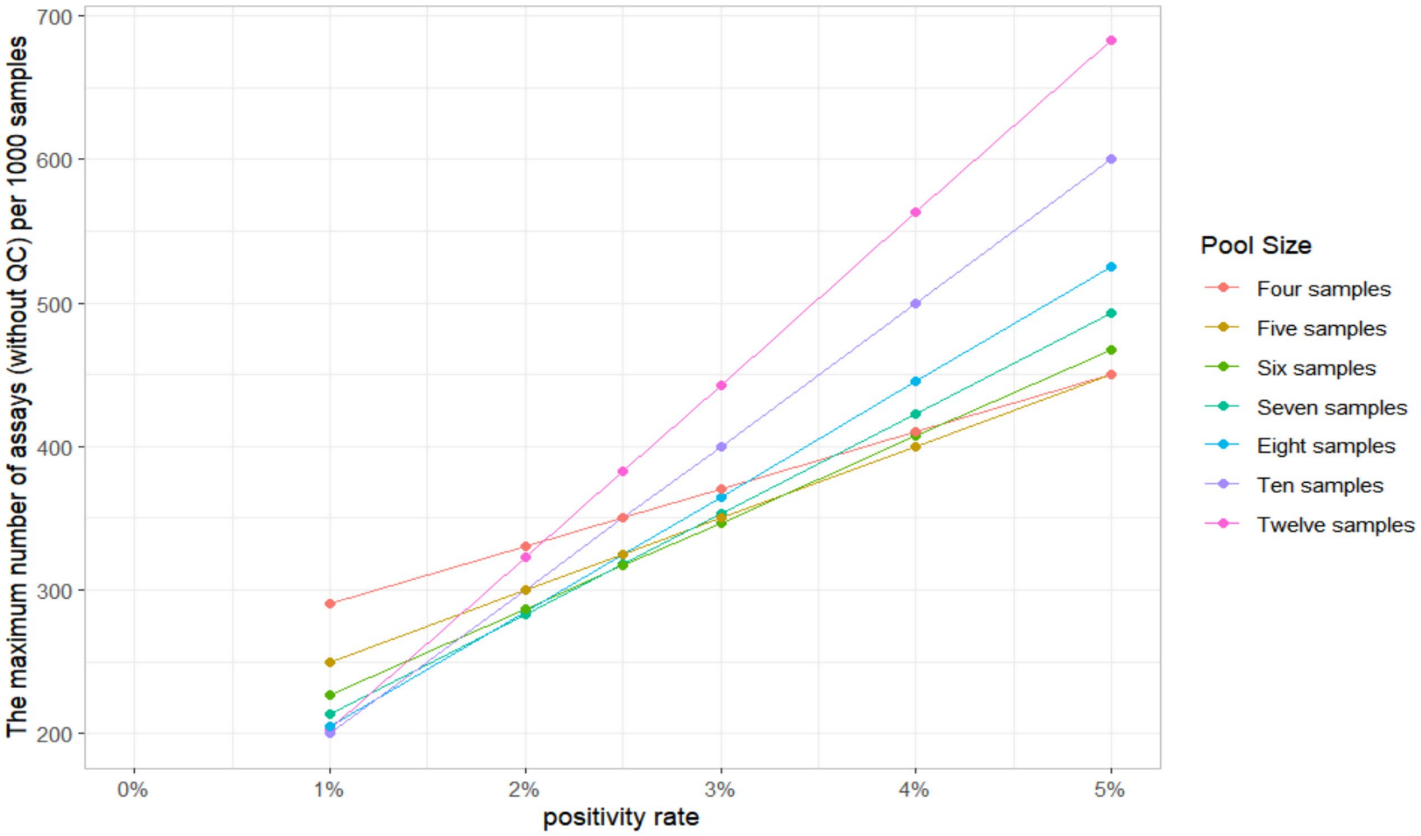
Maximum number of tests required per 1000 samples based on disease positivity rates.

When the 11 positive urine samples were diluted according to the specified ratio, the detection rates varied from 50 to 90.91%. The lowest detection rates were in the 3, 7, 8, and 9 pool size, and the highest rates were in the 4 and 5 pool size. The detection results of different sample pool sizes are shown in Table 1. The positive rate of the population in this study was 2.3%, and the result of the optimal sample pool formula was between 7 and 8. The optimal sample pool sizes corresponding to different positive rates are shown in Table 2. The maximum number of tests (without QC) for the 1,000 samples will vary according to the positivity rate, the size of the sample pool, and the efficiency of the sample pool will peak at approximately 6–8 sample size (Table 3; Figure 2). The selection of 6 sample size (5:1) as the appropriate pool size was ultimately determined by a combination of cost–benefit and sensitivity considerations for detecting losses. This pool size was subsequently scaled up to all positive samples to validate the study design and ensure robust detection perform.

**Table 1:** Results of different sample pool sizes (*n* = 11).

Pool size	Initial Ct value (single test)	Repeat Ct value	2 samples	3 samples	4 samples	5 samples	6 samples	7 samples	8 samples	9 samples	10 samples
Q1	27.9	27.6	27.9	28.2	29.0	29.6	29.6	29.6	30.3	30.2	30.6
Q2	28.4	28.8	29.4	30.0	30.7	30.7	31.0	31.1	31.8	31.6	31.5
Q3	38.8	37.9	37.4	38.9	38.9	37.0	39.7	-	-	-	-
Q4	40.2	39.6	36.7	-^a^	-	38.2	-	-	-	-	-
Q5	35.4	31.9	33.2	33.4	34.1	34.0	34.4	34.5	/^b^	/	/
Q6	36.3	34.2	34.7	34.5	35.2	35.1	35.4	35.6	/	/	/
Q7	35.8	34.8	35.0	36.4	37.1	36.9	36.7	37.0	/	/	/
Q8	37.8	36.3	37.6	36.9	36.8	38.1	38.3	37.5	/	/	/
Q9	37.4	36.4	38.2	38.8	37.7	39.1	38.9	40	/	/	/
Q10	38.3	37.7	-	-	38.9	37.9	38.4	37.6	/	/	/
Q11	36.8	39.1	-	-	38.6	-	-	-	/	/	/
Detection rate		11/11	9/11	8/11	10/11	10/11	9/11	8/11	2/4	2/4	2/4

a Indicates a negative test result.

b When the pre-test is conducted in batches, the pre-test showed a low detection rate in the 8–10 pool size, so no further mixing of the 8–10 pool size is conducted in the subsequent test and therefore no results are obtained.

**Table 2:** Optimal mixing ratios at different positivity rates.

Positivity rate	Optimal sample size
0.2%	22
1.0%	10
1.8%	8
2.5%	7
3.5%	6
5.0%	5
7.5%	4

**Table 3:** The maximum number of tests for the 1,000 samples with positive rate, pool size.

Positive rate	12 samples	10 samples	8 samples	7 samples	6 samples	5 samples	4 samples
5.00%	683	600	525	493	467	450	450
4.00%	563	500	445	423	407	400	410
3.00%	443	400	365	353	347	350	370
2.50%	383	350	325	318	317	325	350
2.00%	323	300	285	283	287	300	330
1.00%	203	200	205	213	227	250	290

Inclusive of initial pool testing and deconstruction of positive pools.

### 3.2 Expanded sample validation results

A total of 8,142 urine samples were collected from participants of the PM/PP health check from January to May 2024, of which 184 (Sample Number P1–P186) were positive, yielding a positivity rate of 2.26%. Of the 184 urine samples, 38.0% were from males, 59.2% were from females, and 2.8% had missing gender information. The age range of 52.51% of the patients was 20–29 years and 48.57% were 30–40 years, with a mean age of 29 years. The mean Ct value of the initial single test was 34.3 (32.0–36.6), the mean Ct value of the single retesting was 32.9 (30.6–34.9), and in three cases, the results turned negative after retesting. Registration code was used to obtain a mixed PN1-PN186 positive sample, of which 175 successfully tested positive and 9 results were negative, for a total of 178 cases that matched the single test results. The results of single and sample pool tests are shown in Table 4. The sensitivity was 96.7% (175/181, 95% CI: 94.1–99.3%), the specificity was 100% (3/3), and the total agreement rate with the single test was 96.7% (178/184, 95% CI: 94.2–99.3%). The PPV was 100% (175/175), and NPV was 33.3% (3/9). The mean ΔCt for single and sample pool tests was 2.8 (2–3.2), *p* < 0.05.

**Table 4:** The results of single and sample pool tests (*n* = 184).

Sample pool test	Single test		Total
	+	–	
+ ^a^	175 (95.1%)	0 (0.0%)	175
– ^b^	6 (3.3%)	3 (1.6%)	9
Total	181	3	184

a Indicates a positive test result.

b Indicates a negative test result.

### 3.3 Discordant samples processing results

6 of the 184 mixed samples did not match the single test results, with Ct values ranging from 37.5–39. Three other positive single samples turned negative after retesting, with Ct values ranging from 38.7–40. After retesting 6 samples with discordant results, three mixed samples (PN155, PN157, and PN160) turned positive, and one (PN166) turned positive after replacement of negative media. Three samples that were tested individually turned positive after retesting. However, only one sample (P175) remained negative after retesting and replacing the negative media.

### 3.4 Results of the analysis of the economic benefits of the sample pool

In this study, using cobas *C. trachomatis* test reagents, it was estimated that the number of people screened and the number of people screened positive would increase threefold compared to single test, assuming that 10,000 test reagents were purchased at a *C. trachomatis* positivity rate of 2.3%. The cost of reagents per capita and the time taken to perform the test are both substantially reduced, as shown in Table 5.

**Table 5:** The economic benefits of sample pool.

Performance Metrics	Single test	6-sample pool test
Number of reagents procured	10,000	10,000
*C. trachomatis* positivity rate	2.3%	2.3%
Number of people screened^a^	10,000	32,258
Relative detection rate	100%	96.7%
Number of positives	230	717
Reagent cost capita	$9.75/case	$2.78/case
Testing time capital^b^	2.6 min/case	0.9 min/case

a 2.3% positivity rate, based on consumption of 31 test reagents per 100 persons.

b Equipped with a fully automated mixing system, excluding QC and based on full boards on the machine.

## 4 Discussion

We present an initial assessment of sample pooling for *C. trachomatis* screening in pre-marital and pre-pregnancy (PMPP) health checks in Shenzhen, China. Preliminary validation of the feasibility of sample pooling test for *C. trachomatis*. Particularly when assayed with a pool of 6-mixed samples, it enables significant savings in reagents, assay reagents, and increased screening within acceptable PCR sensitivity losses.

The mixing pool is configured using negative specimens as diluents, so a urine specimen that is positive on a single test will have a reduced concentration of pathogens (10). The PCR results also showed some increase in Ct values as expected, with a mean ΔCt value of 2.8 (2–3.2), *p* < 0.05. The sensitivity of the pooling test was reduced compared to single test, but test efficiency increased with pool size. If the sample pool size exceeds a certain threshold, the number of assays for mixing pool deconstruction will increase, leading to a decrease in assay efficiency. In this study, a 6-sample pool was ultimately selected for nucleic acid assay, resulting in an estimated savings of 69.53% in assay reagents compared to the survey by S Morré SA et al. (18). Peeling et al. (19) showed that mixing five male archival urine samples for PCR resulted in a sensitivity of 94.4%. Kacena et al. (20) showed a sensitivity of 100% for the determination of a 4-urine sample pool from females and 98.4% for a 10-urine sample pool, and in the present study, not differentiating between genders, the overall sensitivity of the sample pool test is 96.7% (95% CI:94.1–99.3%). According to the ‘Guidelines for Technical Review of the Registration of *C. trachomatis* and/or *Neisseria gonorrhoeae* Nucleic Acid Tests’ (21), for commonly used sample types (e.g., urethral and cervical swabs), the number of *C. trachomatis*-positive samples must be ≥150, or the lower limit of the 95% CI for both the positive compliance rate and overall compliance rate must exceed 90%. The results of this study fully met these regulatory requirements (22).

In this study, the samples with inconsistent results were retested again, and the 6 samples with positive single-test pool-test negatives (ct:37.5–39) and the 3 single-test samples with positive initial retest negatives (ct:38.7–40) were all weakly positive. Conversion of mixed specimens to negative may be due to mixing, concentration below the lower limit of detection, or the presence of inhibitors in negative specimens causing conversion of otherwise weakly positive specimens (23). Inconsistent results of a single sample may be due to degradation of the nucleic acid fragments over a long period, resulting in a lower concentration than the lower limit of detection (24, 25).

In the practical application of the sample pool test, if the pool test is positive, further testing of individual samples must be performed separately, increasing the workload of laboratory staff, so this study included the length of testing in the benefits analysis for a more comprehensive evaluation of pool test (26). In addition, this study also showed that the pool resulted in a small number of weakly positive patients being missed, with a miss rate of 3.3% (95% CI: 0.71–5.92%). However, Quan Zou et al. (27) show that 23.9% of participants are found to have spontaneous clearance of *C. trachomatis* naturally within a median time of 27 days, with a final result of conversion to negative. Dukers-Muijrers NHTM et al. (28) studied the follow-up of chlamydia-infected individuals and assessed clearance by routine quantitative PCR and active PCR tests. Carson Klasner et al. (29) also discuss the self-clearance of *C. trachomatis* and point out knowledge gaps and future research directions. The present study showed that most of the missed detections occurred in weakly positive samples and were also more likely to be self-cleared. It is shown that the use of gold nanoparticles (Au NPs) and microbial assay enrichment pre-treatment can lead to a significant increase in the detection rate of positive samples at low concentrations (30–32).

This study has several limitations. First, as a reverse-validation study conducted with known positive samples, further validation is needed using routine screening samples with unknown test results to fully evaluate the strategy’s feasibility in real-world settings. On this basis, we will comprehensively collect the basic characteristics of the tested population and results of other relevant diagnostic indicators, in order to investigate the variations in sensitivity, specificity, and other diagnostic measures across different subgroups. Second, before widespread implementation of the pooling strategy can be promoted, a standardized operational protocol must be developed through multi-laboratory collaboration, addressing critical aspects such as optimal pool size, sample processing procedures, and quality control measures. Finally, while the sensitivity reduction was modest (3.3%), primarily affecting samples with high Ct values, the clinical implications for detecting low-burden infections and spontaneously clearing cases require further investigation through outcome studies.

## 5 Conclusion

In conclusion, our study demonstrated the great feasibility of the sample pool screening strategy for *C. trachomatis*, especially the 6-sample pool can better balance the detection efficiency and accuracy, and can save a lot of detection time, reagent cost, etc., with acceptable loss of sensitivity. Therefore, the application of the sample pool strategy will be beneficial for the screening of a larger PM/PP population. This study provides valuable insights into the pool detection of *C. trachomatis* in China and provides theoretical support for the improvement of screening strategies.

## Data Availability

The original contributions presented in the study are included in the article/Supplementary material, further inquiries can be directed to the corresponding author.

## References

[1] Chen X Peeling RW Yin Y Mabey DC. The epidemic of sexually transmitted infections in China: implications for control and future perspectives. BMC Med. (2011) 9:111. doi: 10.1186/1741-7015-9-111, PMID: 21975019 PMC3203037

[2] WHO. (2025) Chlamydia. Available online at: https://www.who.int/news-room/fact-sheets/detail/chlamydia. [Accessed March 5, 2025].

[3] Yue X Gong X Li J Zhang J. Epidemiologic features of genital *Chlamydia trachomatis* infection at national sexually transmitted disease surveillance sites in China, 2015-2019. Chin J Dermatol. (2020) 53:596–601. doi: 10.35541/cjd.20200317, (in Chinese)

[4] Molano M Meijer CJ Weiderpass E Arslan A Posso H Franceschi S . The natural course of *Chlamydia trachomatis* infection in asymptomatic Colombian women: a 5-year follow-up study. J Infect Dis. (2005) 191:907–16. doi: 10.1086/428287 15717266

[5] Lanjouw E Ouburg S de Vries HJ Stary A Radcliffe K Unemo M. European guideline on the management of *Chlamydia trachomatis* infections. Int J STD AIDS. (2015) 27:333–48. doi: 10.1177/0956462415618837 26608577

[6] Yao H Li C Tian F Liu X Yang S Xiao Q . Evaluation of *Chlamydia trachomatis* screening from the perspective of health economics: a systematic review. Front Public Health. (2023) 11:1212890. doi: 10.3389/fpubh.2023.1212890, PMID: 37881345 PMC10595018

[7] Grobe N Cherif A Wang X Dong Z Kotanko P. Sample pooling: burden or solution? Clin Microbiol Infect. (2021) 27:1212–20. doi: 10.1016/j.cmi.2021.04.007, PMID: 33878507 PMC9477502

[8] Ben-Ami R Klochendler A Seidel M Sido T Gurel-Gurevich O Yassour M . Large-scale implementation of pooled RNA extraction and RT-PCR for SARS-CoV-2 detection. Clin Microbiol Infect. (2020) 26:1248–53. doi: 10.1016/j.cmi.2020.06.009, PMID: 32585353 PMC7308776

[9] Dorfman R. The detection of defective members of large populations. Ann Math Stat. (1943) 14:436–40. doi: 10.1214/aoms/1177731363

[10] Chong BSW Tran T Druce J Ballard SA Simpson JA Catton M. Sample pooling is a viable strategy for SARS-CoV-2 detection in low-prevalence settings. Pathology. (2020) 52:796–800. doi: 10.1016/j.pathol.2020.09.005, PMID: 33036772 PMC7508550

[11] May S Gamst A Haubrich R Benson C Smith DM. Pooled nucleic acid testing to identify antiretroviral treatment failure during HIV infection. J Acquir Immune Defic Syndr. (2010) 53:194–201. doi: 10.1097/QAI.0b013e3181ba37a7, PMID: 19770802 PMC2915780

[12] Sun HY Chiang C Huang SH Guo WJ Chuang YC Huang YC . Three-stage pooled plasma hepatitis C virus RNA testing for the identification of acute HCV infections in at-risk populations. Microbiol Spectr. (2022) 10:e0243721. doi: 10.1128/spectrum.02437-21, PMID: 35499354 PMC9241589

[13] Sethi S Roy A Garg S Venkatesan LS Bagga R. Detection of *Chlamydia trachomatis* infections by polymerase chain reaction in asymptomatic pregnant women with special reference to the utility of the pooling of urine specimens. Indian J Med Res. (2017) 146:S59–63. doi: 10.4103/ijmr.IJMR_981_15 29205197 PMC5735572

[14] Currie MJ McNiven M Yee T Schiemer U Bowden FJ. Pooling of clinical specimens prior to testing for *Chlamydia trachomatis* by PCR is accurate and cost saving. J Clin Microbiol. (2004) 42:4866–7. doi: 10.1128/JCM.42.10.4866-4867.2004, PMID: 15472365 PMC522303

[15] Kapala J Copes D Sproston A Patel J Jang D Petrich A . Pooling cervical swabs and testing by ligase chain reaction are accurate and cost-saving strategies for diagnosis of *Chlamydia trachomatis*. J Clin Microbiol. (2000) 38:2480–3. doi: 10.1128/JCM.38.7.2480-2483.2000 10878029 PMC86948

[16] Regen F Eren N Heuser I Hellmann-Regen J. A simple approach to optimum pool size for pooled SARS-CoV-2 testing. Int J Infect Dis. (2020) 100:324–6. doi: 10.1016/j.ijid.2020.08.063, PMID: 32866638 PMC7455250

[17] Black MS Bilder CR Tebbs JM. Optimal retesting configurations for hierarchical group testing. J R Stat Soc Ser C Appl Stat. (2015) 64:693–710. doi: 10.1111/rssc.12085 PMC4495770 26166904

[18] Morré SA Meijer CJ Munk C Krüger-Kjaer S Winther JF Jørgensens HO . Pooling of urine specimens for detection of asymptomatic *Chlamydia trachomatis* infections by PCR in a low-prevalence population: cost-saving strategy for epidemiological studies and screening programs. J Clin Microbiol. (2000) 38:1679–80. doi: 10.1128/JCM.38.4.1679-1680.2000, PMID: 10747169 PMC86525

[19] Peeling RW Toye B Jessamine P Gemmill I. Pooling of urine specimens for PCR testing: a cost-saving strategy for *Chlamydia trachomatis* control programmes. Sex Transm Infect. (1998) 74:66–70. doi: 10.1136/sti.74.1.66, PMID: 9634309 PMC1758079

[20] Kacena KA Quinn SB Howell MR Madico GE Quinn TC Gaydos CA. Pooling urine samples for ligase chain reaction screening for genital *Chlamydia trachomatis* infection in asymptomatic women. J Clin Microbiol. (1998) 36:481–5. doi: 10.1128/JCM.36.2.481-485.1998, PMID: 9466763 PMC104564

[21] Peris MP Alonso H Escolar C Tristancho-Baró A Abad MP Rezusta A . Detection of Chlamydia trachomatis and *Neisseria gonorrhoeae* (and its resistance to ciprofloxacin): validation of a molecular biology tool for rapid diagnosis and treatment. Antibiotics. (2024) 13:1011. doi: 10.3390/antibiotics13111011, PMID: 39596706 PMC11591347

[22] Chinese Society of Dermatology, National Center for STD Control, Chinese Center for Disease Control and Prevention, China Dermatologist Association, Chinese Association Rehabilitation of Dermatology. Guideline for the diagnosis and treatment of *Chlamydia trachomatis* urogenital infection in China. Chin J Dermatol. (2024) 57:193–200. doi: 10.35541/cjd.20230712

[23] Schachter J Hook EW Martin DH Willis D Fine P Fuller D . Confirming positive results of nucleic acid amplification tests (NAATs) for *Chlamydia trachomatis*: all NAATs are not created equal. J Clin Microbiol. (2005) 43:1372–3. doi: 10.1128/JCM.43.7.1372-1373.2005 15750110 PMC1081269

[24] Vojtech L Paktinat S Luu T Teichmann S Soge OO Suchland R . Use of viability PCR for detection of live *Chlamydia trachomatis* in clinical specimens. Front Reprod Health. (2023) 5:1199740. doi: 10.3389/frph.2023.1199740, PMID: 37601895 PMC10436598

[25] Kohlhoff S Roblin PM Clement S Banniettis N Hammerschlag MR. Universal prenatal screening and testing and *Chlamydia trachomatis* conjunctivitis in infants. Sex Transm Dis. (2021) 48:e122–3. doi: 10.1097/OLQ.0000000000001344, PMID: 33346588

[26] Hogan CA Sahoo MK Pinsky BA. Sample pooling as a strategy to detect community transmission of SARS-CoV-2. JAMA. (2020) 323:1967–9. doi: 10.1001/jama.2020.5445 32250394 PMC7136853

[27] Zou Q Xie Y Zhang L Wu Q Ye H Ding Y . Spontaneous clearance of *Chlamydia trachomatis* and its associated factors among women attending screening for chlamydia in Shenzhen, China. Int J Infect Dis. (2024) 149:107269. doi: 10.1016/j.ijid.2024.107269, PMID: 39413961

[28] Dukers-Muijrers NHTM Janssen KJH Hoebe CJPA Götz HM Schim van der Loeff MF de Vries HJC . Spontaneous clearance of *Chlamydia trachomatis* accounting for bacterial viability in vaginally or rectally infected women (FemCure). Sex Transm Infect. (2020) 96:541–8. doi: 10.1136/sextrans-2019-054267, PMID: 32066588

[29] Klasner C Macintyre AN Brown SE Bavoil P Ghanem KG Nylander E . A narrative review on spontaneous clearance of urogenital *Chlamydia trachomatis*: host, microbiome, and pathogen-related factors. Sex Transm Dis. (2024) 51:112–7. doi: 10.1097/OLQ.0000000000001905, PMID: 38290156 PMC11017733

[30] Kellogg JA Seiple JW Klinedinst JL Levisky JS. Improved PCR detection of *Chlamydia trachomatis* by using an altered method of specimen transport and high-quality endocervical specimens. J Clin Microbiol. (1995) 33:2580–3. doi: 10.1128/jcm.33.10.2580-2583.1995 PMC228572 8567922

[31] Shao L Guo Y Jiang Y Liu Y Wang M You C . Sensitivity of the standard *Chlamydia trachomatis* culture method is improved after one additional in vitro passage. J Clin Lab Anal. (2016) 30:400–5. doi: 10.1002/jcla.21932 PMC6807024 26987564

[32] Moradi F Delarampour A Nasoohian N Ghorbanian N Fooladfar Z. Recent advances in laboratory detection of *Chlamydia trachomatis* using gold (au) nanoparticle-based methods; another evolution of nanotechnology in diagnostic bacteriology. Microchem J. (2024):111373. doi: 10.1016/j.microc.2024.11137

